# Alterations in intestinal Archaea composition in pediatric patients with Crohn’s disease based on next-generation sequencing – a pilot study

**DOI:** 10.1080/19490976.2023.2276806

**Published:** 2023-11-13

**Authors:** A. Krawczyk, T. Gosiewski, B. Zapała, K. Kowalska-Duplaga, D. Salamon

**Affiliations:** a Department of Molecular Medical Microbiology, Division of Microbiology, Jagiellonian University Medical College, Krakow, Poland; b Department of Pharmaceutical Microbiology, Jagiellonian University Medical College, Krakow, Poland; c Jagiellonian University Hospital in Krakow, Krakow, Poland; d Department of Pediatrics, Gastroenterology and Nutrition,Jagiellonian University Medical College, Krakow, Poland

**Keywords:** Archaea, archaeome, Crohn’s disease, NGS, inflammatory bowel disease; molecular microbiology

## Abstract

Intestinal dysbiosis can lead to the induction of systemic immune-mediated inflammatory diseases, such as Crohn’s disease Although archaea are part of the commensal microbiota, they are still one of the least studied microorganisms. The aim of our study was the standardization of the optimal conditions and primers for sequencing of the gut archaeome using Next Generation Sequencing, and evaluation of the differences between the composition of archaea in patients and healthy volunteers, as well as analysis of the changes that occur in the archaeome of patients depending on disease activity. Newly diagnosed patients were characterized by similar archeal profiles at every taxonomic level as in healthy individuals (the dominance of *Methanobacteria* at the class level, and *Methanobrevibacter* at the genus level). In turn, in patients previously diagnosed with Crohn’s disease (both in active and remission phase), an increased prevalence of *Thermoplasmata*, *Thermoprotei*, *Halobacteria* (at the class level), and *Halococcus*, *Methanospaera* or *Picrophilus* (at the genus level) were observed. Furthermore, we have found a significant correlation between the patient’s parameters and the individual class or species of Archaea. Our study confirms changes in archaeal composition in pediatric patients with Crohn’s disease, however, only in long-standing disease. At the beginning of the disease, the archeal profile is similar to that of healthy people. However, in the chronic form of the disease, significant differences in the composition of archaeome begin to appear. It seems that some archaea may be a good indicator of the chronicity and activity of Crohn’s disease.

## Introduction

The human gut microbiome is composed of a large number of microorganisms. Bacteria constitute the largest population and are the most common subject of human microbiome research,^1^ although recently more and more attention has been paid to fungi, viruses, and protozoa.^2–5^ Intestinal microbiota plays an important role in human physiological functions, through metabolic activity, interaction with the immune system, protection against pathogens, or participation in the digestion of food.^6–8^ Symbiotic relationships between microorganisms and host may be disturbed by the influence of disruptive factors related among others, with the abuse of antibiotics and non-steroid anti-inflammatory drugs,^9^ inappropriate lifestyle and diet rich in simple carbohydrates and emulsifiers,^10,11^ smoking,^12^ excessive stress^13^ or pathological conditions.^14^ All of these can lead to intestinal dysbiosis primarily involving reduction of beneficial bacteria and the growth of pathogens which can trigger chronic diseases, including Crohn’s disease (CD).^15^

Crohn’s disease, along with ulcerative colitis (UC), belongs to the main two types of inflammatory bowel disease (IBD) which manifest as chronic inflammation in the digestive tract. The exact mechanisms leading to the initiation of CD are not entirely clear; however, it is presumed to be associated with an inappropriate host immune response to intestinal dysbiosis in a genetically predisposed individual. The growing evidence suggests that correction of this abnormality might help control inflammation in patients^16,17^ so identifying the key alternations in the composition of microorganisms that contribute to inflammation appears to be crucial in the development of targeted therapeutic approaches. Several species of fungi and bacteria have been postulated to play a role in the triggering CD, including *Candida tropicalis*,^18^ *Malassezia* spp.^19,20^ *Mycobacterium paratuberculosis*,^21,22^ *Bacteroides fragilis* ^23^ or adherent-invasive *Escherichia coli* (AIEC),^24,25^ However, as yet there is no definitive proof of a specific etiological factor in IBD.

Archaea are one of the least studied members of the gut microbiota and for many years their participation in the study of the human microbiome was overlooked. However, several single studies have recently proposed their possible role in IBD. Scanlan et al. showed lower prevalence of methanogens in fecal samples from patients with IBD compared to the control group.^26^ In turn, Chechoud et al. documented that children had lower archaeal colonization than adults but alterations in the archaeal composition were not related to IBD.^27^ Another independent study reported substantial metabolic changes in a patient with episodic colonic inflammation that were associated with the evolution of the disease as a direct consequence of alterations in the intestinal microbiota composition, notably involving archaea.^28^ Blais-Lecour et al. showed that *Methanospharea stadtmanae* were more common in IBD patients in comparision to the healthy volutnteers.^29^ Interestingly, researchers have demonstrated that these patients developed higher levels of plasma-specific IgGs compared to healthy control subjects. Moreover, it has been shown that *M. stadtmanae* can induce the strong response by secreting the proinflammatory cytokine TNF in peripheral blood mononuclear cells.^29^ Considering a study conducted by Bang et al. which demonstrated that human immune cells stimulated by *M. stadtmanae* activated the release of high level of TNF-α and interleukin 1β, it can be concluded that archaea have great immunogenic potential, which may play a significant role in CD.

Archaea are difficult to detect, and thus the human archaeome is not well characterized, so it is hard to determine whether these microorganisms may play relevant role in health and disease. Archaea are similar in shape and size to bacteria, but they carry out other metabolic processes and possess different genes than prokaryotes. Moreover, archaeal membrane is unique and is composed of stable glycerol-ether lipids. These bonds are highly chemically resistant and help Archaea survive in extreme environments.^30^ In 1966, methane was detected in human breath^31^, and this encourages researchers to study feces and isolate the microorganisms responsible for methane production. In this way, the first archaea in the human digestive tract, belonging to the methanogens group, were discovered.^32^ Subsequently, the development of molecular methods based on 16S rDNA provided evidence for the presence of other genus of archaeons in human biological materials, including *Thermoplasmata*, *Haloarchaea* or *Sulfolobus*. ^33,34^ Variations in the presence and abundance of detected archaea based on 16S rDNA analysis are divergent and related to differences in DNA isolation protocoles, the use of different primer pairs and testing methods.^30^ To date, there is only one comprehensive study about archeal composition in human digestive tract, including 1,167 nonredundant archaeal genomes recovered from human samples collected across 24 countries and rural and urban populations which shows how many species of archaea reside in the digestive tract and emphasizes how crucial further research focusing on the analysis of archaea in individual disease entities may be.^35^

Such studies will allow a more reliable determination of the relationship between microorganisms in the gastrointestinal tract and the pathogenesis and course of many diseases (among others CD).

The aim of our study was to optimize the amplification conditions (including pairs of primers) for preparation of genomic library used in next generation sequencing (NGS) for the analysis of the intestinal ‘archaebiome’ and to evaluate the differences between the composition of archaea in CD patients and healthy children, as well as the changes that occur in the archeobiome of patients depending on disease activity.

## Materials and methods

### Patients

Children and adolescents aged 2 to 18 years were recruited for the study in the years 2016–2020 at the Univeristy Children’s Hospital in Krakow. The diagnosis of CD was made according to the Porto criteria^36^ based on clinical manifestations, endoscopy, histology, and radiology. Disease activity was determined based on the pediatric Crohn’s disease activity index (PCDAI). All experimental protocols were approved by the Jagiellonian University Bioethics Committee (No. 122.6120.67.2015 and 1072.6120.21.2020). All procedures performed were in accordance with the Declaration of Helsinki. Informed consent was obtained from patients’ parents or legal guardians (for all patients under 18 years of age) and, additionally, from patients themselves if they were above 16 years old.

We conducted our research based on the principles of case-control study. Children were divided into two main groups according to PCDAI.

1. Pediatric patients with active CD, PCDAI > 10 points. In this group, patients were classified into two subgroups: NeD-CD – newly diagnosed CD patients (before the implementation of any treatment), *n* = 50; and – AC-CD – active, previously diagnosed and treated CD, *n* = 16.
2. Pediatric patients with non-active CD who were in clinical remission during inclusion in the study – Rem-CD (PCDAI ≤10 points), *n* = 39.

The use of antibiotics, probiotics or antifungal drugs 30 days before enrollment, confirmed infection of the gastrointestinal tract, or the presence of an isolated perianal fistula were exclusion criteria.

Healthy, nonhospitalized children and adolescents (*n* = 40) aged 2 to 18 years who had not used antibiotics, probiotics, or antifungal drugs within 30 days prior to stool sample collection and without IBD family members, were recruited into the control group.

### Samples

The materials subjected to the analysis were stool samples collected from individuals qualified for the study. The samples were immediately frozen at − 80°C at the University Children’s Hospital in Krakow, then delivered in deep freeze conditions to the Division of Microbiology of Jagiellonian University Medical College, Kraków, Poland. At the same time, fecal calprotectin and blood biochemical parameters (blood count, sedimentation rate, albumin, protein, glucose, iron) were assessed. All analyses were performed in specialized laboratories at the University Children’s Hospital according to medical diagnostic procedures.

### Isolation of DNA

The stool samples were thawed and archaeal DNA was extracted from 150 mg of each patient’s stool sample. We used a universal method developed by us to extract the DNA of microorganisms (both bacteria and archaea). Isolation was performed, using the Genomic Mini AX Stool (A&A Biotechnology, Gdynia, Poland) along with pretreatment, including enzymatic lysis using lysozyme (A&A Biotechnology), lysostaphin (A&A Biotechnology) and mutanolysin (A&A Biotechnology), followed by mechanical lysis using a FastPrep homogenizer (MP Biomedicals). Briefly, 150 μl NaCl, 20 μl of lysozyme, 10 μl of lysostaphin and 5 μl of mutanolysin were added to the tubes containing feces and glass homogenizer beads. The samples were homogenized (30 s, speed 4.0 m/s) using FastPrep and incubated for 30 min at 37°C. The consecutive steps of DNA isolation were performed according to the Genomic mini AX Stool protocol pursuant to the producer’s instructions. After isolation, DNA concentration and purity were verified spectrophotometrically using a NanoDrop instrument (Thermo Fisher Scientific, Boston, MA, USA).

### Optimization of amplification conditions (including pairs of primers) for NGS sequencing to study the composition of intestinal archaea

A reference sample consisted of a mixture of DNA from the following archaea: *Methanospharea stadtmanae* (DSM 3091), *Methanosacrinia* spp. (DSM 805), *Methanobrevibacter smithii* (DSM 861), *Halorubrum* spp. (DSM 11366). *M. stadtmanae*, and *M. smithii* are the most frequently described archaea in the human digestive tract, therefore, we wanted to be sure that these species would be detected and correctly assigned during the bioinformatic analysis. On the other hand, *Halorubrum* belongs to a different class of archaea, so we wanted to get a diverse control sample to make the analysis reliable and target different archaea to ensure that the primers used would be able to detect other species that may have appeared in the patients’ feces. Negative control (containing sterile water nucleic acids free) was included throughout the experimental procedure and NGS sequencing. A PCR reaction was prepared in four runs, testing four different pairs of primers in order to select the most optimal amplification conditions and obtain the best taxonomic coverage of archaea (Table 1). We have tested four different pairs of primers (previously described in four independent publications) in order to obtain the best taxonomic coverage of archaea. Trial reactions were carried out on a standard sample containing a mixture of DNA from different archaea to provide an objective analysis.

**Table 1.** Primer pairs used during the PCR amplification of archaeal communities.

Pair of primers	Sequences 5’ -> 3’	References
I	F: AATTGGAKTCAACGCCKGR R: TGTGTGCAAGGAGCAGGGAC	^27^
II	F: ATTAGATACCCSBGTAGTCC R: GGCCATGCACYWCyTCTC	^37^
III	F: ACGGGGYGCAGCAGGCGCGA R: GGACTACVSGGGTATCTAAT	^38^
IV	F: CCCTAYGGGGYGCASCAG R: GGACTACVSGGGTATCTAAT	^39^

Adapter sequences for the MiSeq sequencer (Illumina, San Diego, USA) were included with each pair of primers. The adapter sequences were the following^40:^

F: TCGTCGGCAGCGTCAGATGTGTATAAGAGACAG

R: GTCTCGTGGGCTCGGAGATGTGTATAAGAGACAG

The reaction conditions were varied (Table 2) and then the effectiveness of the amplification was verified during electrophoretic separation on 1.5% agarose gel (Prona, Basica Le, Burgos, Spain). The FastGene FAS – Digi PRO imaging system (Nippon Genetics, Duren, Germany) was used for the visualization of the product of a PCR. The thermal cycling conditions included an initial denaturation at 95°C for 5 min followed by 40 cycles of 95°C for 30 s, 55°C for 30 s, and 72°C for 30 s. Next, NGS was performed on the basis of the protocol and MiSeq (Illumina) sequencing platform.^40^ Based on the analysis of obtained results, optimal experimental conditions and optimal primer sequences were selected to further study of patient samples.

**Table 2.** Composition of the reaction mixture for PCR.

Reaction component	Volume (μl)		
	Ist test	IInd test	IIIrd test
Water (A&A Biotechnology)	8.5	7.5	6.5
Kapa polymerase (Roche, Wilmington, USA)	12.5	12.5	12.5
Forward primer (Genomed, Warsaw, Poland)	0.5 (10 mM)	0.5 (10 mM)	0.5 (10 mM)
Reverse primer (Genomed, Warsaw, Poland)	0.5 (10 mM)	0.5 (10 mM)	0.5 (10 mM)
Archaeal DNA	3	4	5

### Preparation of genomic library on patient samples

The DNA isolates from the patients samples were amplified in thermocycler (T100 Thermal Cycler, BioRad, California, USA) with the most optimal primers pair selected from those listed above in Table 1. Reaction was performed based on primers complementary to the highly conserved fragment of 16S rRNA which is unique to a given microbial species. V regions of the 16S rRNA gene were amplified with primers specific for the region, which included the sequences of MiSeq Illumina flow adapters. The primers also included overhang adapter sequences at the 5’ end enabling the addition of labeling and adaptor sequences. Then, the PCR products were purified using AmpliClean Kit (Nimagen, Nijmegen, Netherlands). Next, index PCR was prepared using the Nextera XT index kit (Illumina San Diego, California, United States). During the IInd PCR, amplicons were labeled by attaching the indicator sequences – indices. At this stage, appropriately prepared DNA fragments were used consisting of the sequences of primers, the sequences of which correspond to DNA sections attached at an earlier stage (overhang adapter), labeled fragments (indices) and end sections – adapters (P5 and P7), which allowed carrying out the later stages of the NGS process. This step allowed to double label each amplicon with a unique combination of N and S indicators in order to quickly identify every sample following the end of sequencing. The samples were purified once more. Next, libraries were fluorescently measured using the Quant-iT PicoGreen dsDNA Assay Kit (Thermo Fisher Scientific), normalized to 10 nM and pooled with 30% spiked PhiX control DNA (Illumina)

Then, the sample containing 96 pooled barcoded samples was applied to the cartridge (MiSeq Reagent Kit v3 600 cycles, Illumina) and the NGS was performed using the MiSeq sequencer (Illumina). Automated cluster generation and paired-end sequencing with a 13-cycle index read was carried out.^40^

### Bioinformatics analysis

The raw sequencing reads, generated as FASTQ files, were analyzed using the 16S Metagenomics workflow version 1.1.1 (Illumina). In the first analysis step, the paired-end reads (2 × 300 bp) were trimmed with 0.05 (corresponding to a Phred quality score Q ≥ 14) quality scores. Then, adapter trimming based on the Nextera XT V2 adapters as references was performed. The taxonomic classification of 16S rRNA targeted amplicon reads was made using a taxonomic database, the Ribosomal Database Project (RDP) Classifier, a nave Bayesian classifier. The true biological sequences were generated from raw sequencing reïads using Divisive Amplicon Denoising Algorithm 2 (DADA2). The 16S rRNA sequences were classified into the new higher-order taxonomy and assignments from domain to genus. The representative sequences, based on sequence similarities and assigned taxonomy to them, were grouped into operational taxonomic units (OTU) and then clustered into groups. Furthermore, the generated OUTs were manually aggregated by their taxonomic names according to Integrated Taxonomic Information System (ITIS) online database.^41^ The proportion of mapped reads was calculated by summing up the total read count for each taxon and dividing it by the total mapped reads of the given sample. The percentage of the total abundance was calculated by dividing the combined abundance of the species by the total reads in OTUs. All statistical analyses and graphs were performed using PAST Software (v 4.11)^42^ except for diagrams presenting the correlations of archaeons with clinical and biochemical parameters created with the visplore web application.^43^ Differences between study groups, in terms of the number of reads assigned, the number of identified phylum, class, and genera, and their relative abundances, were tested using a rank sum Friedman test. Wilcoxon signed ranks test was performed to compare the relative abundance of archaeons at different taxonomic levels between study groups. A significance alpha = 0.05 was used. To compare biological diversities across samples and between study groups, diversity profiles were calculated for archaeal communities based on information regarding the similarities between species in the community which was taken into account by a matrix Z. The similarity matrix incorporates information about genetic similarity, phenotypic similarity, or any other biologically meaningful source of similarity between two or more entities. This method of diversity calculation is not influenced by rare or abundant taxa. The diversity profiles plot was generated using PAST v.4.11. Due to the fact that some representatives of Archaea were highly dominant, despite diversity indices calculation, the dominance index was also estimated, which gives the abundance of the most abundant species. It was calculated as the sum of the relative abundance of the most abundant taxa, or a special case of relative dominance with rank 2.

A thorough analysis of metagenomic data was performed according to successive bioinformatic tasks involving the following steps: 1. raw sequencing data, in FASTQ files format collection, 2. quality control and selection of high-quality reads, 3. assembly of larger coherent sequence constructs (contigs), 4. the detection of open reading frames (ORFs) in genomic areas, containing gene encoding sequences, 5. gene annotation (the measuring of genes’ homology), 6. taxonomic analysis (sorting raw sequencing reads taxonomically and phylogenetically), 7. comparative integrative analysis.

## Results

### The concentration and purity of DNA

The following purity criteria for the isolated DNA were assumed: A260/A280 ≥ 1.8; < 2.0. All samples examined met this criterion. The DNA concentration oscillated between 150–270 ng/µl.

### Optimization of amplification conditions

The size of the PCR reaction products (expected ~550 bp) was verified during electrophoretic separation and visualized in the FastGene FAS – Digi Pro (Nippon Genetics, Duren, Germany) in the presence of UV light. The bands were obtained at the appropriate size and lengths in all samples (according to the positive control attached to each reaction). However, in the case of primer pairs No. 1, 3 and 4, separation of tested samples resulted in fainter bands compared to the products obtained with primer pair No. 2. In addition, in several samples, bands appeared in unexpected places, which indicated the presence of nonspecific products. Therefore, based on electrophoretic separation, primer pair no. 2 was considered as the most optimal. The most optimal reaction mixture included: 3 μl of DNA, 12.5 μl of Kapa polymerase, 0.5 μl of each primer, and 8.5 μl of water (i.e. Ist test in Table 2). The thermal conditions were as follows: 95°C for 5 min followed by 40 cycles of 95°C for 30 s, 55°C for 30 s, and 72°C for 30 s.

### The characteristics of the group

There were 105 pediatric CD patients and 40 healthy children included in the study. The anthropometric and clinical characterization of the examined groups is presented in Table 3.

**Table 3.** Anthropometric and clinical characteristics of the studied groups.

	NeD-CD	AC-CD	Rem-CD	Control
	n = 50	n = 16	n = 39	n = 40
**General characteristics**				
Age (yrs); mean (± SD)	13 (±2.9)	15 (±2.8)	12.5 (±4.3)	11 (±4.1)
Female:Male (ratio)	21:29 (0.72)	8: 8 (1)	17:22 (0.77)	25:15 (1.6)
BMI (kg/m^2^; mean (± SD)	17.0 (±3.1)	18.1 (±2.7)	18.1 (±3.2)	18.3 (±3.8)
PCDAI; mean (± SD)	34.4 (±12.7)	38.2 (±18.6)	4.1 (±3.9)	N/A
**Biochemical parameters**				
Glucose (mmol/l); mean (± SD)	4.6 (±0.8)	5.1 (±0.6)	4.9 (±0.8)	N/A
Iron (µmol/L); mean (± SD)	6.7 (±4.5)	9.7 (±7.4)	11.3 (±7.4)	N/A
Protein (g/L); mean (± SD)	68.9 (±8.3)	72.8 (±7.6)	76.2 (±7.0)	N/A
**Pharmacological treatment**				
**(number of patients)**				
5 – ASA	N/A	16	35	N/A
AZA	N/A	8	30	N/A
Corticosteroids	N/A	0	4	N/A

5-ASA 5-aminosalicylic acid; AZA– azathioprine; PCDAI– Pediatric Crohn’s Disease Activity Index.

NeD-CD – active, newly diagnosed CD; AC-CD – active, previously diagnosed CD; Rem-CD – remission of CD.

### Analysis of next-generation sequencing results performed on reference samples with archaeal DNA

The highest number of reads (at every taxonomic level) and the best matches with the reference sample were obtained with primer pair no. II (Table 4). Thus, based on this analysis, primer pair no. II was selected to study patients archeobiome.

**Table 4.** Number of reads at given taxonomic level, using four different pairs of primers.

Taxonomic level	Pairs of primers			
Number of reads				
**Phylum**	I	II	III	IV
*Euryarchaeota*	26989	110910*	68481	32926
*Crenarchaeota*	0	2*	0	2*
**Class**	I	II	III	IV
*Methanobacteria*	13561	51803*	34922	15727
*Halobacteria*	10865	47934*	27100	13718
*Methanomicrobia*	2550	11021*	6440	3474
*Methanococci*	1	10*	8	0
**Genus**	I	II	III	IV
*Methanobrevibacter*	10354	39468*	28666	12362
*Halorubrum*	10854	47778*	27087	13712
*Methanosphaera*	3202	12221*	6236	3356
*Methanosarcina*	2548	11005*	6425	3472

*the highest number of reads for a given taxonomic level of *Archaea*.

### Analysis of the biodiversity of the gut archaeabiome in study populations

**Figure 1.**
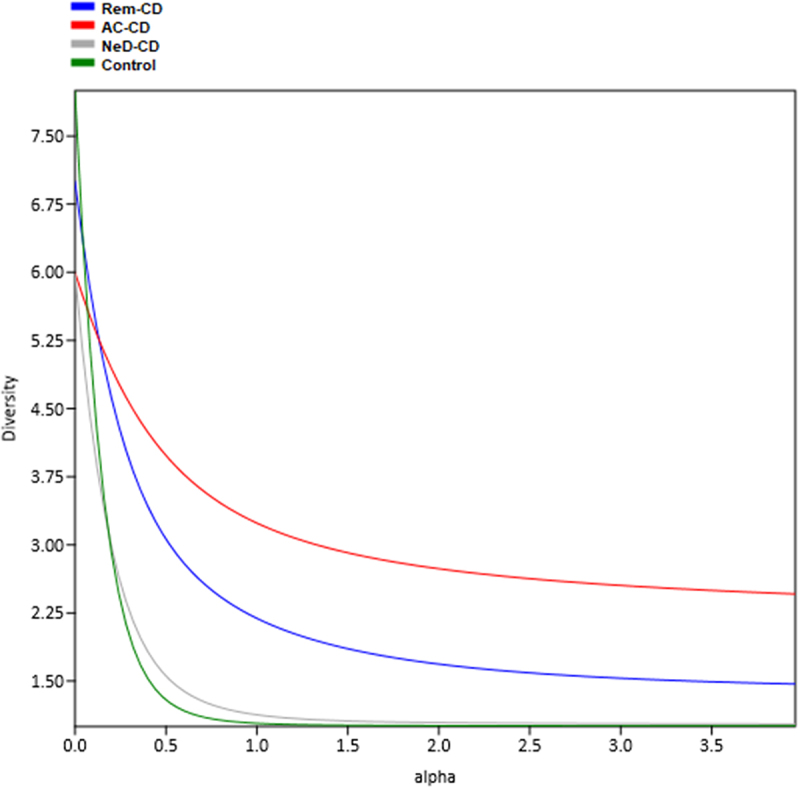
The plot presents alpha diversity profiles together and the magnitude of differences in diversity between study groups. Each study group was assigned a different color of the curve. The higher the curve is, the higher the diversity. If the profiles cross, it means that the diversities are non-comparable. This calculation was made for all datasets analyzed, incorporating phylogenetic diversity as a measure of taxa similarity and naive calculations.

The highest alpha diversity was observed in group AC-CD (red curve in Figure 1) as well as in group Rem-CD (blue curve), while the lowest diversity was characterized for groups NeD-CD and control.

NeD-CD – active, newly diagnosed CD; AC-CD – active, previously diagnosed CD; Rem-CD – remission of CD.

No statistically significant differences were observed at the phylum level. At the class level, statistical differences were noted between groups NeD-CD and AC-CD (*p* =.003). Furthermore, we documented significant differences between groups: NeD-CD vs. Rem-CD (*p* =.0001), Rem-CD vs. control (*p* =.0001), AC-CD vs. control (*p* <.01), and NeD-CD vs. control (*p* <.01) at the genus level.

Analysis of beta diversity at the class level showed statistically significant differences between the groups: NeD-CD and AC-CD (*p* <.01). At the phylum and genus level, there were no statistically significant differences.

### Analysis of the composition of gut archaea in the studied groups

The intestinal archaeabiome was evaluated at five taxonomic levels (phylum, class, order, family, genus). Due to the limited availability of archaeal databases, analysis at the species level (L7) was encumbered with shortcomings and the number of reads was insufficient for objective analysis; therefore, we focused on describing the results to the genus level.

**Figure 2.**
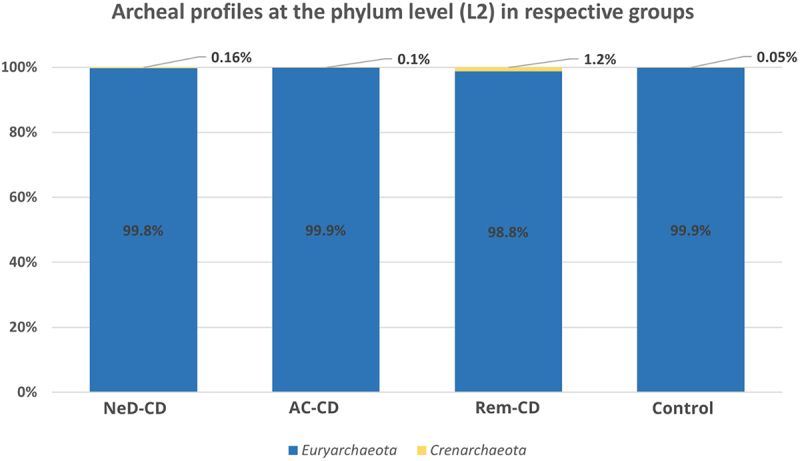
Percentage composition of archaea at the phylum level.

*Euryarchaeota* consituted the dominant phylum in each study group, respectively: 99.8% in Ia, 99.9% in Ib, 98.8% in II and 99.9% in the control (Figure 2). At this level, we did not observe statistically significant differences.

NeD-CD – active, newly diagnosed CD; AC-CD – active, previously diagnosed CD; Rem-CD – remission of CD.

**Figure 3.**
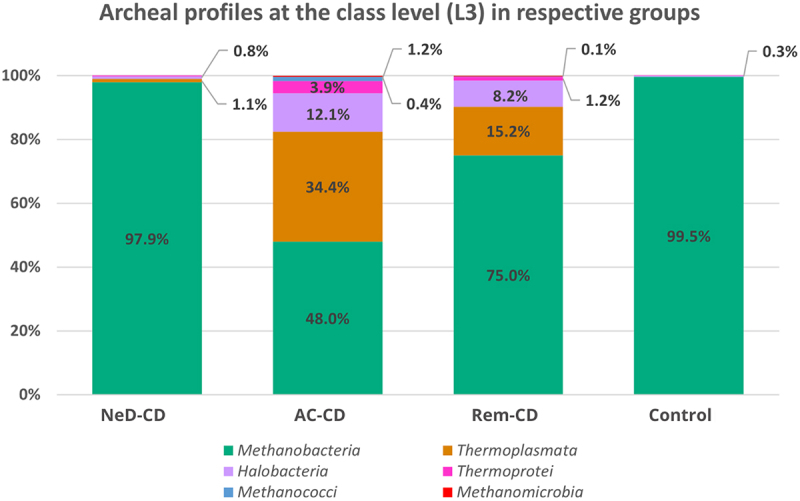
Percentage composition of archaea at the class level.

At the class level (Figure 3), *Methanobacteria* dominated in all groups, however, a clearly lower percentage of these archaea was observed in group AC-CD (48%) compared to groups: NeD-CD (97.9%, *p* <.001), II (75%, *p* =.015), or control (99.5%, *p* <.001). Moreover, in groups AC-CD and Rem-CD a quite high abundance of *Thermoplasmata* was noted (34.4%; 15.2%, respectively) compared to NeD-CD (1.1%) or the control (0.03%). Statistically significant differences were documented between NeD-CD and Rem-CD (*p* =.005), NeD-CD and AC-CD (*p* <.001), AC-CD and Rem-CD (*p* =.006), AC-CD and control (*p* <.001), Rem-CD and control (*p* =.005). We also observed an increased abundance of *Halobacteria* in groups AC-CD (12.1%) and Rem-CD (8.2%) compared to NeD-CD (0.8%) or control (0.3%). Statistically significant differences were documented between NeD-CD and Rem-CD (*p* =.02), NeD-CD and AC-CD (*p* =.002), AC-CD and control (*p* <.001), and Rem-CD and control (*p* =.02). The percentage of *Thermoprotei* was higher in groups AC-CD (3.9%) and Rem-CD (1.2%) compared to the other groups (0.16% in NeD-CD, 0.05% in control); however, these differences were not statistically significant.

NeD-CD – active, newly diagnosed CD; AC-CD – active, previously diagnosed CD; Rem-CD – remission of CD.

**Figure 4.**
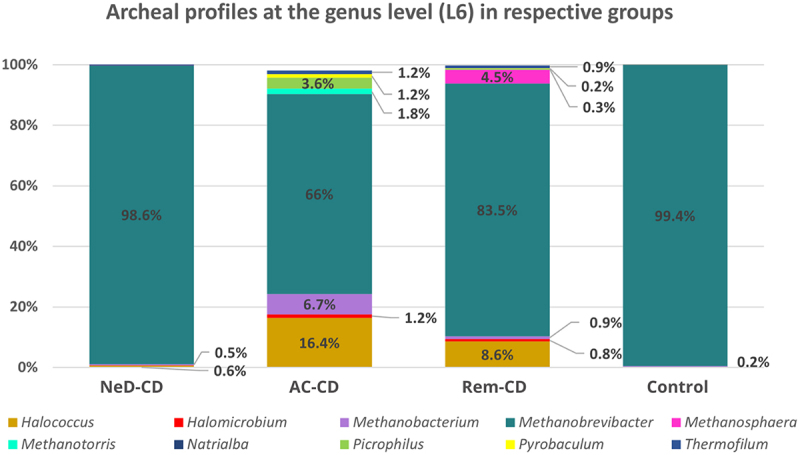
Percentage composition of archaea at the genus level.

The genus *Methanobrevibacter* was the most dominant taxon, representing 98.6% of all sequences in NeD-CD; 66% in AC-CD; 83.5% in Rem-CD, and 99.4% in the control (Figure 4). A statistically lower level of this genus was documented in group AC-CD compared to NeD-CD (*p* =.013) and control (*p* = 0.01). Interestingly, in groups AC-CD and Rem-CD, there was a higher percentage of *Halococcus* (16.4%; 8.6%, respectively) compared to NeD-CD (0.6%, *p* <.05) and control (0.2%, *p* <.05). In addition, *Picrophilus* (3.6%) and *Methanobacterium* (6.7%) represented a relatively large percentage in group AC-CD, while in group Rem-CD, we observed a higher prevalence of *Methanospharea*; however, these changes were not statistically significant.

NeD-CD – active, newly diagnosed CD; AC-CD – active, previously diagnosed CD; Rem-CD – remission of CD.

### Correlation between Archaea and clinical parameters of patients

Patients’ clinical parameters, i.e. PCDAI; calprotectin; BMI; age; glucose/iron/protein levels and the applied treatment were correlated with the individual class and genus of Archaea. We took into account the correlations of each type of treatment with individual archaea. Both 5-ASA monotherapy (1), azathioprine monotherapy (2), or corticosteroids monotherapy (3) were included to the analysis, as well as combination treatment including all combinations applied by patients (1 + 2, 1 + 2 + 3, 2 + 3). None of the used treatments showed correlation with the composition of archaea.

**Figure 5.**
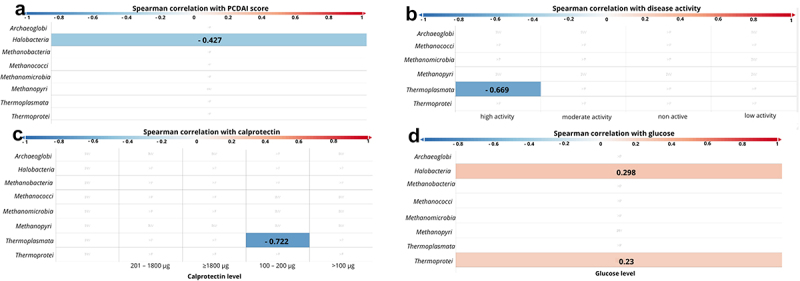
Correlation between class of archaea and patients’ clinical parameters. The strength of the correlation is displayed by color ranges from blue to red. Blue colors indicate negative correlation, red colors indicates positive correlation. The more intense the color, the strongest correlation. If the r-value is displayed on the bar, it means that the correlation was statistically significant.

At the class level, statistically significant a negative correlation was observed between the PCDAI score and *Halobacteria* (*p* < 0.001, *r* = − 0.427; Figure 5a). When the patients were divided into four groups based on the degree of disease activity according to PCDAI (i.e. „non active”: < 10 points, „low activity” 11–25 points, „moderate activity” 26–50 points, and „high activity” > 51 points, Figure 5b) we also found a negative correlation between ‘high active disease’ and *Thermoplasmata* (*p* = 0.024, *r* = − 0.669; Figure 5b). A negative correlation has also been documented between *Thermoplasmata* and calprotectin levels (*p* =.028, *r* = − 0.722; Figure 5c). Furthermore, a positive correlation of the glucose level with *Halobacteria* (*p* =.002, *r* = 0.298) and *Thermoprotei* (*p* =.021, *r* = 0.23, Figure 5d) was observed.

Correlation between class of archaea and : A – PCDAI score; B – disease activity; C – calprotectin; D – glucose;

PCDAI – pediatric Crohn’s disease activity index.

**Figure 6.**
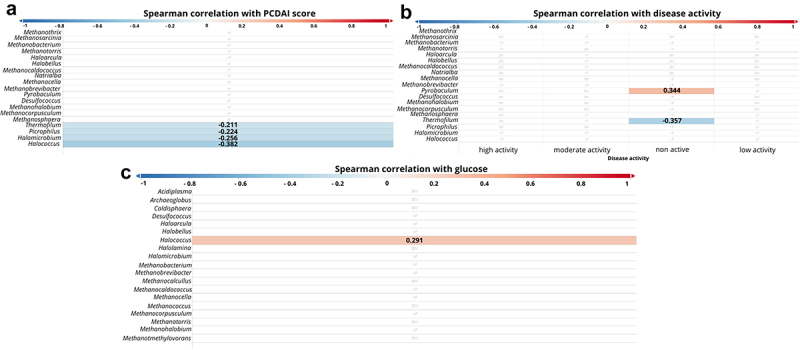
Correlation between genus of archaea and patients parameters. The strength of the correlation is displayed by color ranges from blue to red. Blue colors indicate negative correlation, red colors indicates positive *correlation*. the more intense the color, the strongest correlation. If the r-value is displayed on the bar, it means that the correlation was statistically significant.

At the genus level, statistically significant negative dependencies were observed between PCDAI and the following archaeons: *Thermofilum* (*p* =.035, *r* = − 0.211), *Picrophilus* (*p* =.025, *r* = − 0.224), *Halomicrobium* (*p* =.010, *r* = − 0.256), *Halococcus* (*p* =.001, *r* = − 0.382, Figure 6a) and positive *Pyrobaculum* (*p* =.004, *r* = 0.344, Figure 6b). Furthermore, we observed a positive correlation between glucose level and *Halococcus* (*p* =.003, *r* = 0.291, Figure 6c).

Correlation between genus of archaea and : A – PCDAI score; B – disease activity; C – glucose;

PCDAI – pediatric Crohn’s disease activity index.

There was no statistically significant correlation of age, sex, BMI, or applied treatment with individual Archaea (both at the class and phylum level).

## Discussion

Taking into account that bacterial^15,44,45^ and fungal^46,47^dysbiosis is commonly observed among patients with IBD, we performed the analysis of intestinal Archaea in pediatric CD patients to verify whether there are also abnormalities in the composition of archaeons. While the importance of bacteria in Crohn’s disease has been, up to now, a common subject of research, there are only few studies considering the contribution of Archaea.^29,48^ In addition, available studies are very limited and do not take into account the correlation between biochemical parameters of patients or the phase of disease activity and archaeons. To our knowledge, there is only one publication that extensively describes the correlation of many microbial species (including archaea) with metabolic and biochemical changes over time.^28^

Moreover, we have tested four pairs of primers in order to select the most appropriate pair to prepare genomic libraries for sequencing the gut archaeobiome and to increase sensitivity of the PCR reaction. The high number of reads and the compliance of the taxonomic composition with the tested reference sample made it possible to select the best sequences for the study with patients. We have selected the bioinformatics methods depending on the data obtained, which ensured a reliable analysis. For the DNA isolation procedure, we use our universal method developed for DNA extraction of bacteria, and archaea. Although lysozyme is not effective against archaea, it certainly had no negative effect on archaea present in the samples.

We noticed that across the samples and in the study groups, the community diversity greatly altered regarding diversity levels, thus to calculate archaeal community diversity, we decided to create diversity profiles. As we know, the validity of comparing diversities may be criticized due to the arbitrary choice of a diversity index.^41^ In several samples, we noticed a larger number of taxa while the others had a larger Shannon index. When we calculated the Dominance-D index for several samples was about 1, showing one taxon dominates the community completely. The calculated Dominance-index was about 1 representing greater dominance. Because the dominance index is negatively correlated with alpha diversity indices, we may speculate that more dominant communities of Archaea are less diverse. For this reason, we defined a family of diversity indices dependent upon a single continuous parameter. To be sure that the diversity ordering was robust, we compared a number of diversity indices by creating diversity indices profiles using PAST v.4.11 software. In that way, we could consider taxa similarity information, effective diversity, or multiple diversity metrics, providing insights into microbial datasets that were not detectable with classical univariate diversity metrics. Creating a diversity profile enabled us to provide information about the effects of rare and highly abundant species on diversity calculations which we noticed regarding very low reads of archaeal taxon across the samples.

In this study, we enrolled pediatric patients with different stages of CD (both active and in remission), as well as healthy children in the control group. The study participants were unrelated and were from the same geographical region. The patients in all groups were similar in age and BMI. Males slightly predominated in the CD patients group; however, according to epidemiological data,^49,50^ Crohn’s disease in children predominantly affects boys, which was reflected in our studied population.

Our research has shown that archaeal profiles (at every taxonomic level) were similar in newly diagnosed CD patients (at the very beginning of the disease, group NeD-CD) compared to healthy volunteers. However, with the duration of the disease (groups AC-CD and Rem-CD), significant changes occured in the composition of Archaea (Figures 3, 4).

Analysis of alpha diversity (evaluating biodiversity within the studied groups) at the archaeal class and genus level showed that groups AC-CD and Rem-CD were characterized by increased archaeal biodiversity, i.e. a higher number of taxons in a single sample. In turn, in the control group, we documented the lowest archaeal diversity (Figure 1). These are interesting observations, because in the case of the bacteriobiome and mycobiome, opposite trends are observed.^47,51,52^ Based on the obtained results, we can conclude that the homogeneous composition of archaeons (dominance of one or two Archaea) is characteristic of a “healthy” microbiome. However, when there is a long-term inflammatory process in the body, other species of Archaea appear, and biodiversity increases. It is interesting that in patients at the very beginning of the disease (NeD-CD), the biodiversity was at a level similar to that in the control group. This supports the above hypothesis that changes in the archaeome occur with the progression of chronic inflammation, which can be considered a secondary effect of the disease.

Analysis of beta diversity (which is a measure of biodiversity among the study groups) has shown that there were statistically significant differences only at the class level between groups NeD-CD and AC-CD. This may confirm the observation that with the duration of the disease, the diversity of the composition of archaea changes, which is noticeable especially in the case of exacerbation of the disease. The lack of statistical differences between studied groups at the genus level is probably due to the low number of reads in single samples, which limits statistical analyzes of beta diversity. The groups are not comparable in terms of beta diversity.^53^

At the phylum level, the vast majority of sequences annotated as *Euryarchaeota* in all groups (Figure 2). This phylum is highly diverse and includes the most common Archaea (like *Methanogens*, *Halobacteria*).^54^ At the class level, archaea belonging to *Methanobacteria* were dominated in all groups. It is consistent with the current state of knowledge because methanogens dominate in the human gut and produce methane as a metabolic by-product.^55–57^ Interestingly, in groups AC-CD and ReM-CD, we documented a clear presence of *Thermoplasmata*, *Halobacteria*, or *Thermoprotei* in contrast to group NeD-CD or control. Some of the detected Archaea, representatives of the hyperthermophilic such as *Crenarchaeota* or others such as *Halorubrum*, *Halococcus* seem not to be detected in human samples. However, as other researchers, we agree with the hypothesis that they may be present, but to be sure further studies should be performed. In our study, we exclude the sequence matching errors. We optimized primer specific to the Archaea species choosing the pairs with the highest specificity (Table 4). In other studies, the hyperthermophilic Archaea have been detected in human samples. In 2005, Rieu-Lesme F. et al. revealed the presence of some representatives of *Crenarchaeota* in the human intestine microbiota^58^. In independent studies, *Crenarchaeota* also have been detected in the human digestive system.^34,59^ It is also very important to note that, the representatives of halophilic Archaea in the human intestinal mucosa may be related to salty food as their source. In several studies, the species of halophilic Archaea have been identified in human samples such as stool samples, intestinal mucosa, and dental plaques^34,60–62^

In these studies, the authors supposed that the presence of these microorganisms was due to the consumption of refined salts and an array of manufactured food products where large quantities of salt are employed in the preservation process such as salted fish, seafood, hides, pork, sausages, and fish-based sauces^63–65^ It was also reported that the high salt concentration during the fermentation process is sufficient for the development of *Halobacteriaceae archaea*, such as *Halococcus thailandensis*, *Natrinema gari*, *Haloarcula salaria*, and *Haloarcula tradensis*.^65–67^ Takahashi et al.,^38^ have documented the detection of *Thermoplasmata* in pig feces before, which confirms that “unusual” archaea may be present in the intestines of mammals. In turn, Oxley et al.^34^ documented the presence of *Halobacteria* in German patients with IBD. The confirmation of the presence of *Halobacteria* in Polish CD patients in this study provides evidence that these Archaea may be related to IBD, which requires further research. Interestingly, we detected these classes of archaeons in CD patients with long-standing disease (groups AC-CD and Rem-CD), but not in newly diagnosed individuals (NeD-CD). It can be concluded that the presence of these microorganisms is a secondary effect of the disease and is related to the chronic form of CD. Perhaps the conditions that develop in the intestine affected by long-term chronic inflammation (e.g., formation of granulomas, abscesses, depressed scars) favor the promotion of specific Archaea. It should be reminded that people suffering from IBD are characterized by fungal and bacterial dysbiosis. Perhaps long-term changes in fungal and bacterial populations promote the growth of some archaeons, which has been raised in several studies.^68–70^ Basile et al. extensively described the changes in microbial ecology that occur during the progression of the disease (including a significant increase in archaea) and documented how absence of the normally dominant phylum allowed other, rare phyla to dynamically bloom.^28^ Moreover, it was associated with significant metabolic and biochemical changes. These observations are compatible with our findings, in which changes in the archeal composition are clearly visible only in a long-lasting disease.

It may be considered whether the appearance of these Archaea is a secondary effect of the applied treatment; however, we did not show any correlation between the therapy used and the presence of any Archaea, which seems to contradict this hypothesis.

At the genus level, the vast majority of sequences were annotated as *Methanobrevibacter* in all groups. According to the literature, *Methanobrevibacter smithii* is the most common intestinal Archaea and can be detected in 96% of the population. The second most frequently detected methanogen is *Methanosphaera stadtmanae*.^29,55,71^ Interestingly, Blais-Lecours et al.^29^ showed that among patients suffering from IBD, this species is detected significantly more often than in healthy people. In addition, these researchers reported that mononuclear cells stimulated with *M. stadtmanae* produced more than 4-fold higher concentrations of tumor necrosis factor (TNF-α) compared to cells stimulated with *M. smithii*.^29^ These results showed the high immunogenic potential of *M. stadmanae* and suggest that this species may be involved in initiating of inflammation in the course of IBD. However, this hypothesis has not been confirmed so far. Based on results obtained in our study, we assume that the increased prevalence of this Archaea described in IBD patients is only a secondary effect of the disease because in patients with active disease (NeD-CD and AC-CD groups) we did not find an increased abundance of *M. smithii*. Interestingly, this species of archaeon appears in patients in remission (i.e. group Rem-CD). It is likely that the healing of the mucosa and changes in gut conditions during the regeneration process favor the multiplication of *M. smithii*. In addition, patients in nonactive CD are expanding their diet, and intestinal transit is not as rapid as in an exacerbation, which is of great importance for the multiplying of some microrganisms. Chronic diarrhea has previously been suggested to contribute to the loss of slow-growing methanogens that are displaced during conditions of rapid gut transit,^26^ which seems to be confirmed in our study in the absence of *M. smithii* in a group of patients with active CD.

It is interesting that in groups AC-CD and Rem-CD there were “uncommon” genus of Archaea, such as *Halococcus*, *Picrophilus*, *Thermofilum*, or *Methanotorris*, but we suppose that this is a similar cause as we described above when discussing the class level. In our opinion, it is probably the effect of long-term inflammation in the intestines, which favors the colonization of less typical microorganisms. To our knowledge, there is a lack of detailed sequencing of archaeal communities in patients with IBD, so it is difficult to compare our results with others. The few existing studies are based only on PCR^26,29^ or limited sequencing results,^34,72^ which do not include an archaeal profile at this taxonomic level.

Interesting information is provided by the results taking into account the correlations of patient parameters with individual genus of Archaea, obtained in this study. We have shown that archaeons such as *Halobacteria* and *Thermoplasmata* were negatively correlated with PCDAI (Figure 5a) and calprotectin (Figure 5b). This reinforces our consideration that the changes that occur during mucosal healing and the overall improvement of the patient’s intestinal condition may favor the growth of certain Archaea. Worth noting is that glucose levels were positively correlated with the presence of *Halobacteria, Thermoprotei* (Figure 5d) and *Halococcus* (Figure 6c). To our knowledge, this is the first such report and it supports our above considerations that along with expanding patients’ diet (e.g. by providing sugars, the consumption of which is reduced during CD exacerbations), specific Archaea appear in the intestine.

Interestingly, there was no correlation between the age of the patients and the presence of individual archaea. As is well-known, the presence of methanogens increases with age. Their abundance is definitely greater in adults than in children.^73,74^ In our study, all children were quite similar in age, which is probably the reason for the lack of correlation. Of greater importance would be the age correlation of both adult and pediatric patients with archaea; however, in this research we focused only on the study of children and adolescents.

## Conclusions

Due to the lack of research including sequencing of archaeobiome, we had to standardize the selection of optimal primers and PCR conditions for NGS. Moreover, in order to avoid the influence of rare or abundant taxa in determining biodiversity, diversity profiles for archaeal communities were calculated based on information that was included in the similarity matrix. For this reason this work is of a pilot nature.

Our study confirms changes in the archaeal composition in pediatric patients with CD, however, only in long-standing CD. At the very beginning of the disease, there are no abnormalities in the composition of the archaeobiome, and this composition is very similar to that of healthy people. However, in the chronic form of the disease, significant differences in the composition of the intestinal Archaea begin to appear. It seems that some archaeons may be a good indicator of the chronicity and activity of Crohn’s disease. Archaea are very poorly understood microorganisms, and their exact role in the human body is not clear, so more research is needed taking into account this group of microorganisms in order to precisely define their contribution to human health and disease.

## Data Availability

Fastq files are available The Jagiellonian University Repository – online access: https://ruj.uj.edu.pl/xmlui/handle/item/304596?search-result=true&query=FASTQ+files+gosiewski&current-scope=&rpp=50&sort_by=score&order=desc. doi: 10.26106/908a-c237
